# Access to Prenatal Testing and Ethically Informed Counselling in Germany, Poland and Russia

**DOI:** 10.3390/jpm11090937

**Published:** 2021-09-20

**Authors:** Marcin Orzechowski, Cristian Timmermann, Katarzyna Woniak, Oxana Kosenko, Galina Lvovna Mikirtichan, Alexandr Zinovievich Lichtshangof, Florian Steger

**Affiliations:** 1 Institute of the History, Philosophy and Ethics of Medicine, Ulm University, 89073 Ulm, Germany; cristian.timmermann@uni-ulm.de (C.T.); katarzyna.woniak@geschichte.uni-halle.de (K.W.); oxana.kosenko@uni-ulm.de (O.K.); florian.steger@uni-ulm.de (F.S.); 2 Department of Humanities and Bioethics, Saint-Petersburg State Pediatric Medical University, 194100 Saint Petersburg, Russia; glm306@yandex.ru (G.L.M.); humdisc@mail.ru (A.Z.L.)

**Keywords:** non-invasive prenatal testing (NIPT), clinical ethics, pregnancy termination, Eastern Europe, reproductive rights

## Abstract

The development of new methods in the field of prenatal testing leads to an expansion of information that needs to be provided to expectant mothers. The aim of this research is to explore opinions and attitudes of gynecologists in Germany, Poland and Russia towards access to prenatal testing and diagnostics in these countries. Semi-structured interviews were conducted with n = 18 gynecologists in Germany, Poland and Russia. The interviews were analyzed using the methods of content analysis and thematic analysis. Visible in all three countries is a connection of prenatal medicine with the politically and socially contentious issue of pregnancy termination. Respondents in Poland and Russia concentrated on the topic of inadequate resources. Quality of information for expectant mothers is an important point in all three countries. Only in Germany was the issue of language barriers in communication raised. With regard to non-invasive prenatal testing (NIPT) respondents in Germany focused on the ethical issues of routinization of testing; in Poland and Russia they concentrated on fair access to NIPT. Challenges in all three countries arise from structural factors such as imprecise and prohibitive regulations, lack of resources or organization of healthcare services. These should be addressed on a political and medico-ethical level.

## 1. Introduction

The development of reproductive technologies allows us to reduce risks related to late-term births, expanding reproductive choices. Giving birth to the first child at a later age is a worldwide trend. According to the statistics for 2019, women’s mean age for their first child is 31.2 years for Germany, 29.7 years for Poland [1] and 27 years for Russia [2]. Due to changes in the labor market and insufficient regulations to accommodate a balance between work and family life, there is an increasing percentage of women who have children at an older age [3]. This carries additional considerations that need to be evaluated for both the expectant mother and the fetus [4]. It is crucial to be informed about the likelihood of disabilities and rare conditions, as they may increase the need for extensive care during the child’s lifetime. Care activities disproportionally burden women and may oblige them to abandon their careers, hindering progress in achieving equality of opportunities [5].

Currently, the technological progress allows for early detection of genetic mutations in the fetus. Since the 1960s, invasive prenatal diagnostic methods, such as karyotyping, allow for the detection of segmental chromosomal imbalances. Despite their limitations, which include the possibility of miscarriage, invasive genetic diagnostics of the fetus remain widely used [6]. Since commercial introduction in 2011, non-invasive prenatal testing (NIPT) methods revolutionized the field of prenatal medicine by allowing the identification of anomalies without thereby endangering the pregnancy [7]. NIPT uses cell-free fetal DNA present in maternal plasma to assess the risk of fetal genetic abnormalities. The technique is used to detect fetal chromosomal abnormalities such as Down syndrome (trisomy 21), Edward syndrome (trisomy 18), Patau syndrome (trisomy 13) and other aneuploidies, e.g., Turner syndrome, Klinefelter syndrome or Triple X syndrome. NIPT offers an accurate risk assessment for chromosomal abnormalities. A meta-analysis of NIPT accuracy in women at a high risk for fetal aneuploidy reported sensitivity of 99.7, 97.8 and 95.8% for detection of trisomy 21, 18 and 13, respectively. Corresponding clinical specificities are 99.9, 99.9 and 99.8% for trisomy 21, 18 and 13, respectively [8]. Thus, NIPT reduces the need for invasive testing procedures and lowers the probability of procedure-related miscarriages. Notwithstanding its imperfections, including false positive and false negative results, the popularity of NIPT worldwide and its application is increasing rapidly [9,10]. The field is increasing the range of factors that are tested [11].

Of themselves, technological developments in prenatal testing do not necessarily lead to better access to prenatal healthcare. Access to data from prenatal testing does not equate to providing an adequate prenatal diagnosis, which should also involve adequate patient information about possible therapies and interventions. Several factors, such as normative regulations, organization and accessibility of the healthcare services, allocation of resources to prenatal medicine or the social situation in a country affect access to prenatal diagnostics and counselling. Furthermore, some health professionals who provide counselling are not up to date with the new diagnostic methods and in utero treatment options [12]. In other cases, counsellors may not follow the principle of non-directiveness and give too little weight to new ethical developments that include insights from disability studies and feminist perspectives [13]. Due to the rapid technological advancement in prenatal diagnosis and scholarship in medical ethics, counsellors need to continuously inform themselves about the new status of knowledge and patient communication approaches [14].

Uncertainties, invasiveness of some diagnostic procedures, legal barriers over termination of pregnancies, and opportunities for new treatments need all to be communicated to the patient at times of high expectations and pressures [15]. Limits on health care resources affect the availability of the best diagnosis and treatment options, including the availability and quality of counselling. Moreover, the field of prenatal diagnostics is subject to political influence and an object of contention. The strong association of prenatal screening and diagnosis with pregnancy termination leads to pressure from special interests groups on healthcare professionals to invoke the conscientious objection clause and refuse procedures that are against their values or the values of the institutions they work in [16,17]. Ethically appropriate genetic counseling should not only respect the principle of non-directiveness as a commitment not to impose one’s own values on patients [18]. In order to support patients in making autonomous decisions, it should adhere to the ethical principles of beneficence and non-maleficence, especially by considering the individual goals and values of patients, utility of provided information or informational burden placed on the patients.

In Germany, Poland and Russia, NIPT is an add-on to routine prenatal care. In Germany, the Federal Joint Committee decided in August 2021 that from 2022 onwards the test will be offered as a health insurance service and paid from public funds. However, it should only be provided in justified individual cases of elevated risks [19]. Intensive patient information before and after the test is mandatory and a prerequisite for covering costs. In Poland and Russia, physicians have a legal obligation to inform the patient about the nature of genetic prenatal diagnostics, their purpose and the significance of the results. However, public funding is provided only for invasive genetic tests; NIPT is available as a self-pay service. For such services, relevant and appropriate patient information should be provided.

The aim of this research is to examine experiences and attitudes of gynecologists in Germany, Poland and Russia towards access to prenatal testing and counselling in these countries. We interviewed gynecologists working in standard care and specializing in reproductive medicine. The focus of our research concentrated on personal opinions of the interviewees towards (i) current regulations in this area; (ii) the issues of patient information and adequate counselling and (iii) access to novel prenatal testing methods, such as non-invasive prenatal testing (NIPT). These countries were selected for investigation on several grounds. First, they differ in their normative framework concerning prenatal medicine. For example, a restrictive interpretation of conscientious objection or major impediments to pregnancy termination, as recently observed in Poland, raise questions of fair access to prenatal medicine [20,21]. Second, they vary with regard to their economic situation. This influences the allocation of financial resources to healthcare and the accessibility of prenatal diagnostics. Third, they display important differences in their sociocultural demographics, e.g., migration trends contributed to a more ethnically diverse society in Germany than in Poland or Russia. Moreover, influence of conservative social transitions observed in Poland and Russia can affect the practice of patient information towards directiveness of counselling. In contrast, the more liberally oriented social environment in Germany can result in emphasis on patient self-determination during counselling.

## 2. Materials and Methods

We conducted semi-structured interviews with gynecologists in Germany, Poland and Russia. For this research, a qualitative research design in the form of explorative narrative interviews was used. The aim of the qualitative narrative interviews was to gain insight into the subjective views of the interviewees. The formulation of the interview questions and the setting of the focal points aimed to provide statements in the respective context of the interview topic and the background of the interviewees. As a survey method, exploratory interviews offer a certain flexibility by allowing us to ask ad-hoc questions in order to clarify statements or to focus on particularly important issues. This form of interviewing consists of expert questions, narrative parts and focus parts with narrative character [22].

Through online research we have identified the contact data of gynecologists practicing in centers for reproductive medicine or gynecologists with private practices. We have followed purposive sampling to select participants with the potential to provide relevant data pertinent to the research topic [23]. Criteria for selection was to include physicians currently practicing in the field of gynecology, without regard to the size of the practice, age, career path or background. Gender parity among the interviewees was sought. Initial contact with the interview partners occurred by email, in which we introduced the research project and asked for an interview. Gynecologists that responded to our invitation were subsequently contacted via telephone or, at the initial stage of research in Russia, received a written questionnaire. In order to avoid possible biased answers, for example through specific preparation for the interviews, the initial request was only formulated in the general context of “ethical and legal aspects of prenatal medicine”. All interviews were conducted according to the same interview guideline with a catalogue of 12 questions prepared by our multidisciplinary research team. These key questions were formulated on the basis of extensive literature research on all three countries and individual professional expertise of the members of the research team. When necessary, follow-up questions to the main interview themes were asked.

Individual semi-structured interviews were conducted in March and April 2020 in Germany (*n* = 6) and Poland (*n* = 6) and in February and March 2021 in Russia (*n* = 6). All one-on-one interviews were held by telephone. As a result of the COVID-19 pandemic, we first decided to ask questions to Russian healthcare professionals in writing in November 2020. We received *n* = 18 written responses. Due to insufficient data saturation, in February and March 2021 additional one-on-one telephone interviews (*n* = 6) were conducted with Russian healthcare professionals not included in the previous sample. The method of conducting interviews via telephone decreases the influence of setting/site on the participants [24].

The interviews were conducted in the native language of the interviewees, i.e., German, Polish and Russian. The individual interviews lasted between 30 and 60 min. The interviews were conducted by two researchers with a PhD degree and knowledge of the research topic and methods of qualitative research. No previous relationship with the interviewees had been established prior to the study commencement. At the beginning of the interview, the interviewees were informed about the aim and procedures of the research, that their participation was voluntary, about the protection and archiving procedures of the data acquired during the interviews and consent about the publication of results in anonymized form was sought. Because the research questions do not specifically study the influence of individual characteristics of interviewees, i.e., age, gender, career path or professional background, on the research topic, no demographic data were collected during the interviews. In order to provide comparable results from all three countries, the research team decided at the beginning of the study to target six interviews in each country. The issue of sufficient data saturation in regard to the amount and quality of interviews was discussed in the team of the authors.

Interviews were digitally recorded and transcribed by the researchers conducting the interviews. After transcription, the interviews were fully anonymized. Data analysis was conducted using the methods of content analysis [25] and thematic analysis [26,27]. First, the responses of the interviewees were reduced to core elements and statements. These elements were manually coded, extracted and systemized through clustering into main topics and subtopics. These topics were inductively formulated based on the content of the interviews, in order to identify important and recurring themes and differences between responses. Representative quotes, which illustrate various themes, were translated from the language in which the interview was conducted into English. In order to avoid bias, coding and analysis of the interviews were conducted by two researchers not involved in the interviews. For the purpose of triangulation of the results, this process was conducted by these two researchers separately. The results of coding were compared and discussed.

## 3. Results

Interviews focused on three themes: (i) attitudes on current legal regulation of prenatal diagnostics in the country; (ii) views on issues of patient information and access to adequate counselling in prenatal diagnostics; (iii) opinions on access to novel methods of prenatal testing and use of prenatal diagnostics. In the following, we present a narrative synthesis of the interview statements on these four themes. Table 1 shows an overview of thematic focus of the responses in each country. These thematic focus points determine the structure of this section.

### 3.1. Evaluation of Current Regulations

When asked about their assessment of current legal regulations on prenatal diagnostics, interviewees in Germany showed mixed perceptions. The answers concentrated on access to pregnancy termination and the role of physicians. One interviewee indicated that, in this matter, Germany is in the middle, not as restrictive as Eastern European countries, but noticeably not as liberal as France and The Netherlands. One gynecologist condemned German regulations for being far too liberal regarding late pregnancy terminations, particularly after the diagnosis of Down syndrome, sometimes even up to the 40th week, using the term “feticide”. The same physician claimed that the principle of non-directiveness was not respected in prenatal centers—some women were encouraged to abort after the fetus had been diagnosed with Down syndrome. In contrast, one physician categorized German regulations as “rather conservative”. Three of the interviewees had a negative opinion on German regulations for having double standards with their ambiguous phrasing of abortion laws, leaving it too often to physicians to decide on how to proceed, and thereby failing their responsibilities in establishing a clear normative framework. Reference was made to the controversial legal jargon—“unlawful but not liable to prosecution” (“rechtswidrig aber nicht strafbar”)—regulating abortions in Germany.

In Poland, interviewees in their evaluation of legal regulations were mostly deeply concerned with the availability of resources for prenatal diagnostics. Four physicians experienced the lack of health resources as the main hurdle to offering adequate diagnostics. The failure to direct sufficient resources to prenatal diagnostics has a regulating effect, particularly on poorer women who end up excluded from prenatal medicine. Only one physician was satisfied with the regulations and their clarity. One further topic touched upon in the interviews was pregnancy termination. One physician expressed a worry that medical doctors are afraid to offer more than the basic examinations, and were reluctant to propose any further recommendations due to the strong governmental objections to pregnancy terminations and anything which might be associated with the practice. Two physicians suggested that, due to new testing options, the legal limit to offer terminations of pregnancy should be moved from 22 to 24 weeks to allow women enough time to calmly decide on how to proceed.

Four Russian physicians complained about the lack of resources designated to prenatal medicine, which overloaded health professionals and impeded the allocation of sufficient time to each patient. One physician condemned that screening for pregnant women in the third trimester is not offered anymore since the beginning of 2021.

Considering the issue of pregnancy termination, the majority of Russian interviewees called for liberalization of current legislation. Only one interviewee evaluated the regulations as good, and just stated that both physicians and patients need to comply with it. One of the interviewed physicians clearly stated that women’s autonomy in deciding not to have a child with disabilities should be respected, and that the laws protecting such decisions needs to be enforced. Two physicians stated that Russia differs from international legal standards, with one of them even pleading to shift towards these standards. As an example, the interviewee referred to Israel, where, in contrast to Russia, abortion due to fetal malformations with a late manifestation is also possible in the third trimester of pregnancy. Another doctor mentioned, as an example, a case of the prenatal council’s refusal to allow a woman to terminate her pregnancy when her fetus was diagnosed with an agenesis of the corpus callosum, a diagnosis that in European countries is an indication for pregnancy termination.

### 3.2. Patient Information and Access to Adequate Counselling

On the question of adequate patient information in prenatal diagnostics, German interviewees focused on two issues: the availability of information and on information for minority groups with language barriers, e.g., migrants or refugees. On the first issue, the interviewees remarked on the patients’ prior knowledge about the aims and possibilities of prenatal diagnostics. Two gynecologists mentioned that patients are not well informed about the quality of tests and the significance of particular genetic disorders, e.g., Down syndrome. It is particularly difficult for physicians to make patients realize the difference between not finding anything suspicious and of being healthy. Expectant mothers were also unaware about the high rates of abnormalities during pregnancies and their significance. As one interviewee declared, the lack of prior information requires a deep commitment from the counselling physician. In such situations it is difficult to present information in a fully neutral and non-directive manner. Nevertheless, several physicians stated that the availability of online resources is slowly improving patient information. Patients who looked for information on the internet were much better informed. One of the interviewed physicians asks patients to look up the information they post on their webpage before coming to the appointment, so that they have a better idea about the procedure—a practice that, in the view of this interlocutor, offered good results.

The effect and significance of language barriers depended on the physician’s background. Some physicians pointed to language barriers as a major hurdle. One of them said that this was a major problem, going as far as claiming that migrants have a higher proportion of pregnancy complications due to high rates of consanguineous relationships. Another physician pointed out that migrants often refused to include an interpreter. In contrast, one multilingual physician with a migrant background did not consider language barriers a general problem, as often a common language can be found. Only monolingual patients from regions with rare languages are more difficult to reach. Another physician experienced major educational gaps as a greater impediment for providing adequate counselling than language comprehension difficulties alone.

A major issue for Polish gynecologists was misinformation campaigns associating prenatal diagnosis with pregnancy termination. In their view, these hinder the information process and make it difficult to offer adequate treatment options. Women with more conservative values often refused prenatal testing altogether, sometimes without even being open to a conversation about the subject. Physicians condemned this situation, as it impedes them in making the necessary arrangements to deliver complicated births under optimal care conditions in well-equipped facilities.

In addition to the personal values of the patients, Polish interviewees observed that education level constituted an obstacle for adequate information process. One physician observed that this required a major revision of the information process in prenatal medicine to adapt it to the patients’ level of comprehension and their personal values. Nonetheless, two Polish interviewees also observed that patients come with much better knowledge about testing possibilities and their need after reading online materials, in contrast to earlier times. Similarly, as observed in Germany, one of the Polish physicians referred to the extensive materials their practice made available online. However, the same physician remarked that sometimes the overflow of information leads to increased anxiety. As a consequence, many patients are not in a state of mind to clearly discuss and evaluate a diagnosis.

On the role of cultural and language barriers in Poland, one physician pointed that no major barriers exist, since there is basically only one form of mother–child relationship, wanting to know that everything is fine with their offspring. Another physician remarked that patients from migrant backgrounds were aware of the strong legal restrictions in Poland and sometimes were suspicious about health professionals and took extreme measures to escape the system, including going for pregnancy termination at a very late stage to their country of origin.

Concerning the issue of patient information in Russia, the majority of physicians pointed out that patients were offered consent forms with the most frequently requested information. Five physicians observed that some patients already informed themselves through online materials before coming to the practice. However, the patients’ level of education was also perceived as a barrier. One gynecologist mentioned difficulties in explaining to patients the levels of accuracy of the tests. Two interviewees observed that although currently a majority of the patients came better prepared to the practice than in the past, it is much more difficult to explain the diagnosis and its meaning to patients with lower educational levels. Three physicians complained about the lack of opportunities to calmly explain in plain language to the patients the diagnosis and its implications.

Cultural and social differences were generally not considered as obstacles in prenatal practice in Russia. Only one physician observed that people from a Muslim background were more likely to object to termination without being fully prepared to give birth to a child with additional care needs and a complicated medical condition.

In all three countries physicians demonstrated good knowledge of the ethical requirements of seeking informed consent, highlighting the need to give extensive information and actively engage in making this information more accessible. Many physicians took care in avoiding counselling with a directive character, showing concern for protecting patients’ autonomy. Interestingly, ensuring wider accessibility, e.g., by offering counselling to migrants in another language, was not framed as an ethical obligation of physicians towards justice, in the sense of expanding access to healthcare.

### 3.3. Access to Novel Testing Methods

In Germany, differential access to novel testing methods was seen as a challenge, even though the conclusions physicians drew from this inequality varied. One physician welcomed NIPT and claimed that governments need to work to make the least intrusive diagnostics methods widely accessible. In one interview, the lack of access due to financial limitations was seen as an injustice, as it does not allow patients to benefit from technological advancement. In contrast, other interviewees had strong objections against routinization of NIPT. One physician stated that “NIPT is solely there, basically, to filter out Down syndrome children, everything else is a fable” (I2). Two other interviewees condemned the enormous lobby and influence of test manufacturers. They remarked that there is too much advertisement on NIPT, often with a false basis, without taking any responsibilities for inaccuracies or overinflated claims. Commercial interests in diagnostics prevail and governments rarely set up an environment where women have real choices in delivering children with intensive care needs. Another physician argued that new tests do not really change circumstances, as amniocentesis has already been there for three decades. The same physician condemned that many people had the idea that more tests lead to higher rates of success, unconsciously following the belief that by spending more money on tests, they increase the odds that their children are healthy.

In Poland, one interviewee welcomed the use of NIPT, stating that it often calms down anxious and fearful expectant mothers. However, three other physicians complained that NIPT was largely profit-driven, involving often prohibitive costs. Only one physician did not place NIPT a high value, as ultrasound examinations are becoming increasingly sophisticated and able to reveal a large amount of information.

In Russia, physicians judged that the country was not prepared for the increasing demand for the new technological possibilities in prenatal diagnosis and testing. Among the largest complaints were the lack of specialists in the field, increased demand for services and overburdened professionals. Most physicians explicitly mentioned that the branch of prenatal diagnosis was underdeveloped in the country and needed to be established as a priority in the near future. One interviewee in Russia commented on strong variations between the private and public sector. While some tests are offered in the public sector, many more tests are performed in the private sector. In many cases patients themselves proactively seek these tests or already come to the practice with tests performed elsewhere.

Generally, in the case of access to novel testing methods, physicians were aware of the social justice and ethical implications of differential access. While some expected the government to destine more resources to bridge this gap, most physicians actively sought to provide the best prenatal diagnosis they could offer by making the most out of the resources they had. The interviewed physicians where much more likely to consider it as an ethical obligation to expand access with their medical skills than by making use of their interpersonal skills.

## 4. Discussion

The results of the research show that the main challenges in prenatal testing and diagnostics in all three countries do not stem from individual issues between patients and healthcare professionals. They rather arise from structural factors such as imprecise and prohibitive regulations, political and social pressure on patients and physicians, lack of resources or deficient organization of healthcare services.

A major theme in the statements of gynecologists in all three countries is the relationship between prenatal testing and the question of legal termination of pregnancy. In Germany the main concern was the ambiguous regulation on termination of pregnancy. In contrast, evident in Poland and Russia was a call for liberalization of pregnancy termination to facilitate reproductive choices. This demand was explicitly formulated in the responses of interviewees in Poland. Up to January 2021, the termination of pregnancy in this country was possible after a prenatal screening indicating a high probability of severe and irreversible fetal anomaly up to 22 weeks of gestation. In 2020 a survey among gynecologists in Poland performing prenatal diagnostics and testing showed that 46% of respondents approved an extension of access to termination of pregnancy in cases of lethal fetal defects up to the end of gestation [28]. However, the verdict of the Polish Constitutional Court of 22 October 2020 delegalized termination of pregnancy on the basis of lethal fetal defects. Due to societal and political pressure, many healthcare professionals in Poland invoke the conscientious objection clause in cases associated with possible pregnancy termination [29]. This can be associated with political and social pressure. A similar situation is observed in other countries. Professional and social ostracism are factors that influence physicians’ decisions about being involved in pregnancy termination cases [30,31]. Despite ongoing attempts to restrict legislation on abortion, also on the part of the Russian Orthodox Church [32], the laws remain one of the most liberal in the world, since during the first 12 weeks of pregnancy termination can be performed without any restrictions. After 12 weeks, termination is still possible subject to medical considerations.

When asked about their evaluation of current regulations, Polish and Russian interviewees concentrated on the availability of resources, which constrain the access to prenatal medicine. The last survey on the topic by the Polish Supreme Audit Office from 2016 showed that, nationwide, only 19% of women in Poland use prenatal diagnostics. In some regions, this number is as low as 8% [33]. The reason for this situation is underfinancing of prenatal medicine and the geographical location of medical facilities offering publicly financed prenatal diagnostics. In some regions of Poland there are only one or two such centers, which limits physical access to prenatal diagnostics and testing. In Russia, according to official statistics, more than 88% of pregnant women underwent some type of prenatal screening in 2019 [34]. However, in most regions there is no possibility of in-depth diagnosis of hereditary diseases that can be currently identified in the course of prenatal screening. The only centers that have such opportunities are in Moscow, St. Petersburg, Ufa, Tomsk and other large cities. In addition, these tests are mostly paid for by the patients themselves or are not carried out at all.

With regard to the question of patient information, the responses in all three countries involved the issue of adequacy of information provided to patients. Several studies showed that interventions designed to improve the level of information about prenatal screening for Down syndrome have a positive effect on knowledge reception and satisfaction [35]. However, the question of comprehensiveness and the method of information is central. Abundance and variety of information presented in form of probabilities leads to information overload. This constitutes a serious ethical issue, as it may undermine informed consent and self-determined decision-making [36]. In such a situation, the recommendation and advice from healthcare professionals plays an important role for the patient’s decision-making process [37]. In all three study countries physicians had a positive experience with providing patients with online materials for information. Patients who informed themselves over the internet were better prepared and less anxious, two factors that facilitated the information and counselling process. This suggests that a preliminary information session using online materials reduces the risk of overloading the patient with information. Patients could concentrate in the counselling session on getting answers to open questions and seeking further information on their particular situation.

Based on these observations we recommend the provision of online materials as a standard practice in prenatal medicine. In regions with highly restrictive pregnancy termination laws, independent organizations could complement the information provided through governmental channels. Currently, there are good experiences with providing third-party help through telephone hotlines [38]. As some of the interviewed physicians fear that many women do not approach them for prenatal tests due to prejudices and stigmatization, it is important to provide information and establish communication channels that can be accessed anonymously to serve vulnerable groups.

Interviewed physicians in Germany pointed out to the issue of language barriers as a factor influencing access to healthcare. This issue was less important for physicians in Poland and Russia, due to a lower number of patients with migrant backgrounds. Effective communication is key for the provision of quality medicine and has an impact on the level of patient satisfaction [39]. Given the complexity of the topic, difficulties in communication across languages can be seen as a major obstacle in the provision of adequate information in prenatal diagnostics and testing [40]. One possible solution is the use of interpreters, acting not only as translators, but also assuming the role of intercultural mediators. In our study, only one of the interviewees in Germany referred to the use of interpreters in prenatal practice. Involvement of professional interpreters is connected with additional costs and organizational issues. Relaying on ad-hoc interpreters, e.g., family members or healthcare staff is, on the other hand, associated with ethical issues of quality of translation and professional confidentiality [41].

Interviewees in Germany called attention to the question of routinization of prenatal diagnostic methods, especially NIPT. The use of prenatal diagnostic methods, such as ultrasound or amniocentesis, as a standard measure has been observed as an ongoing process in the last decades. The introduction of NIPT changes the ethical questions due to the medicalization of pregnancy and the normalization of prenatal quality control [42]. On the other hand, early detection of trisomies 13, 18, 21 or other genetic disorders is important to prepare expectant mothers and their partners to raise a child with special care needs or to take an informed decision about terminating the pregnancy. Here the issue of high costs of NIPT, raised by interviewees in Poland and Russia, has a direct impact on fair access to the least intrusive prenatal testing method. NIPT is easily applicable, delivers fast test results and avoids the risks associated with invasive testing methods, which may lead to miscarriages. In Germany, the inclusion of NIPT as a publicly funded procedure was extensively debated [43] and is expected to be implemented in 2022 [19]. In Belgium, the Netherlands and the United Kingdom, NIPT has been implemented as part of routine prenatal care [44,45].

## 5. Limitations

The results of this study need to be seen in the light of its limitations. The first limitation is response bias. Gynecologist who were willing to participate in the interviews may have formed unambiguous opinions about the topic of the research. Physicians that refused to participate or did not response to the invitation might have done so because of apprehension to the topic. For this reason, and because of the qualitative nature of this research and the number of conducted interviews could not mirror demographic diversity, the results are not representative and cannot be generalized for the entire gynecologist population in the countries under investigation. Second, the interviews were carried out during a time with major unforeseen social and legal changes. From the beginning of the first interviews in February 2020 until their conclusion in March 2021, the legal regulations in relation to prenatal diagnosis in Poland and Russia changed. These changes may also have affected the opinions of prenatal diagnostic specialists. The verdict of the Polish Constitutional Tribunal of 22 October 2020, which limited the list of cases permitting abortions and an order of the Russian Ministry of Health of 1 January 2021, which removed screening for pregnant women in the third trimester from the free services category, had deep implications for prenatal medicine. As a third limitation, it should be noted that in Russia interviews were mostly carried out in clinics in the urban sector, which may explain the greater availability of prenatal diagnosis for women and the quality of services. Taking under consideration these limitations, further research concerning this research topic is necessary.

## 6. Conclusions

The opinions presented show various focal points of respondents in all three countries. In general, the main challenges in prenatal testing and diagnostics in all three countries do not stem from individual issues between patients and healthcare professionals, but arise from structural factors such as imprecise and prohibitive regulations, lack of resources or deficient organization of healthcare services. For all three countries under investigation, a strong association of prenatal testing and diagnostics with questions of pregnancy termination and a political and social pressure on healthcare professionals who offer such services is observable. This issue was especially noticeable among interviewees in Poland. This can have a serious impact on the accessibility and quality of information. A prenatal consultation that is dominated by prejudices and biased moral and political opinions can have consequences for the life and health of the expectant mother and for the social situation of parents after the birth. Therefore, the ethically appropriate provision of information should occur in a non-directive environment, without political or social pressure exercised on the information process. Moreover, the interviewees in Poland and Russia accentuated the economic component of fair and equitable access to healthcare. Scarcity of resources allocated to this field of medicine should be addressed at the level of healthcare organization in these countries, especially under the context of the changing social attitudes towards family planning. Furthermore, the issue of adequate information for mothers becomes central. The interviewees responses reveal that the increasing complexity of results in prenatal testing puts serious demands on both physicians and expectant parents. Novel methods of patient information, e.g., through verified internet resources or telemedicine, should be considered as a standard part of the information process. Challenges of intercultural communication, as observable in Germany, can impose further obstacles on the information process. With increasing cultural diversity in Poland and Russia, such challenges may also emerge there in the future. Allocating resources for prenatal medicine should include means for professional translators and cultural mediators. Resolution of these challenges cannot occur on the individual level of patient–physician relationship. Political and systemic changes that should be built on fundamental medico–ethical and professional discussions on the aims of prenatal testing are required.

## Figures and Tables

**Table 1:** Thematic focus of the responses in each country.

Country	Thematic Focus of the Responses
	**Evaluation of current regulations**
Germany	Regulation of pregnancy termination
Poland	Availability of resources for prenatal diagnostics Access to pregnancy termination
Russia	Availability of resources for prenatal diagnostics Access to pregnancy termination
	**Patient information and access to adequate counselling**
Germany	Quality of information for patients Provision of information for minority groups with language barriers
Poland	Quality of information for patients Impact of the association of prenatal diagnosis with pregnancy termination
Russia	Quality of information for patients Process of information
	**Access to novel testing methods**
Germany	Routinization of non-invasive prenatal testing Fair access to non-invasive prenatal testing
Poland	Fair access to non-invasive prenatal testing
Russia	Professional competency for the provision of non-invasive prenatal testing Fair access to non-invasive prenatal testing

## Data Availability

The data presented in this study are available on request from the corresponding author. The data are not publicly available due to protection of privacy.
